# Decline of Plasma Concentrations of Interleukin-18 in Severely Malnourished Patients with Anorexia Nervosa: Exploratory Analysis

**DOI:** 10.3390/nu11030540

**Published:** 2019-03-03

**Authors:** Satoshi Tanaka, Tomoko Oya-Ito, Yuki Murakami, Kuniaki Saito, Sho Furuta, Yanjie Yu, Miho Imaeda, Shohko Kunimoto, Norio Ozaki

**Affiliations:** 1 Department of Psychiatry, Nagoya University Hospital, Nagoya 466-8560, Japan; ozaki-n@med.nagoya-u.ac.jp; 2 Faculty of Health and Nutrition, Shubun University, Ichinomiya 491-0938, Japan; tomoko@med.nagoya-u.ac.jp; 3 Organization for Research Initiatives and Development, Doshisha University, Kyotanabe 610-0394, Japan; ymurakam@mail.doshisha.ac.jp; 4 Department of Disease Control and Prevention, School of Medical Sciences, Fujita Health University, Toyoake 470-1192, Japan; saitok@fujita-hu.ac.jp; 5 Nagoya University Graduate School of Medicine, Nagoya 466-8550, Japan; furutasho@med.nagoya-u.ac.jp (S.F.); carmiyu@med.nagoya-u.ac.jp (Y.Y.); kunimot@med.nagoya-u.ac.jp (S.K.); 6 Department of Clinical Oncology and Chemotherapy, Nagoya University Hospital, Nagoya 466-8560, Japan; imaeda-m@med.nagoya-u.ac.jp

**Keywords:** anorexia nervosa, eating disorder, malnutrition, cytokine, interleukin, IL-18

## Abstract

Multiple studies on the dynamics of inflammatory cytokines in patients with anorexia nervosa (AN) have been published, although results are not consistent among reports. Thus the pathophysiologic roles of these cytokines are not clear. We performed an exploratory analysis that included (1) comparisons of plasma interleukin-18 (IL-18) concentrations between patients with AN (*n* = 21) and healthy controls (*n* = 39), and (2) correlations between body mass index (BMI) and IL-18 concentrations in both groups, exploring the relationship between malnourishment and IL-18. Plasma IL-18 levels were significantly decreased in patients with AN compared with controls. Plasma IL-18 levels correlated to BMI in controls, but not in patients with AN. These results suggest that a decline in plasma IL-18 levels in patients with AN is not only due to malnourishment, but other pathophysiologic changes as well. IL-18 has a role in the brain’s reaction to sadness and chronic stress. Therefore, decreased levels of IL-18 may commonly occur in patients with chronic AN.

## 1. Introduction

Anorexia nervosa (AN) is sometimes fatal due to severe malnutrition. Leukocytopenia frequently accompanies AN [1], although the reason for this is unknown. Patients with AN appear to maintain standard immunity and do not show an increased risk for systemic infections [2,3]. Both in vitro and in vivo studies have explored the role of inflammatory cytokines, such as tumor necrosis factor-α (TNF-α), associated with immunity in patients with AN, but findings are inconsistent [2,4,5,6,7,8]. The latest meta-analysis suggests that plasma levels of certain types of inflammatory cytokines (e.g., TNF-α and interleukin-6 (IL-6)), are elevated in a malnourished state of AN and may partially account for the decreased appetite of anorexic patients [9]. However, given its low power and large confidence intervals, whether specific elevated cytokines represent trait or state markers of AN requires further study.

Interleukin-18 (IL-18) is an inflammatory cytokine first identified by Okamura et al. in 1995. It was formerly referred to as an interferon gamma-inducing factor and is known to activate natural killer cells in the spleen [10,11]. IL-18 is found in multiple cell types including immune, hematopoietic, chondrocytic, and intestinal epithelial cells as well as astrocytes and microglial cells in the central nervous system, and is activated through separation from precursors by caspase-1. As a member of the IL-1 family, IL-18 induces inflammation and interferon-gamma production by activation of murine and human T cells in synergy with IL-12, and thus promotes cell-mediated immunity [12]. However, unlike other cytokines, the dynamics of IL-18 in patients with AN have rarely been examined.

In this exploratory analysis, we compared (1) plasma concentrations of IL-18 between patients with AN and healthy controls, and (2) correlations between body mass index (BMI) and IL-18 concentrations in both groups to examine the relationship between malnourishment and IL-18 levels.

## 2. Materials and Methods

The ethics review committees at Nagoya University Graduate School of Medicine and Nagoya University Hospital approved the study protocol, and written informed consent was obtained from all participants.

Patients included 21 Japanese women living in Japan who met the criteria of extremely severe AN (BMI < 15 kg/m^2^) based on the Diagnostic and Statistical Manual of Mental Disorders-5 (DSM-5) [13], including 7 with AN restricting type and 14 with AN binge-eating and purging type. The BMI of patients ranged from 10.34 kg/m^2^ to 14.54 kg/m^2^. Patients’ medical records were reviewed retrospectively for psychiatric comorbidities; two cases had bipolar I disorder, one had bipolar II disorder, one had major depressive disorder, and one had a history of sedative, hypnotic, or anxiolytic use. No other psychiatric comorbidities were identified. Patients were treated in the psychiatric ward of Nagoya University Hospital and received medical nutritional therapy aimed to increase weight by 1 kg per week along with supportive psychotherapy by psychiatrists. They also received psychotropics or plasma electrolyte replenishers, as needed. The control group consisted of 39 healthy participants recruited from medical and co-medical students and hospital staff. A structured interview confirmed the absence of any psychiatric history in the control group. BMI in the control group ranged from 17.12 kg/m^2^ to 33.23 kg/m^2^. Table 1 shows demographic data for both groups.

Blood samples from patients were drawn around 8 a.m., before breakfast. Body height and weight were measured under surveillance in the early morning, before the blood was drawn, every Monday and Thursday, and the data obtained closest to the date of blood draw within the same week were used for analysis. In the control group, blood samples, body height, and weight were acquired during a visit to our institute, so the time of day for sampling was not fixed.

Plasma IL-18 concentrations of all subjects were measured by enzyme-linked immunosorbent assay (ELISA) using a human bioactive IL-18 ELISA kit from Medical Biological Laboratories (Naka-ku, Nagoya, Japan).

All data were analyzed with Excel 2013 (Microsoft Corp., Redmond, WA, USA) and JMP pro 13 (SAS Corp., Cary, NC, USA). The Wilcoxon rank sum test was used to compare plasma IL-18 concentrations between groups. The coefficients (Pearson’s r) and *p*-values were calculated between plasma IL-18 concentrations and BMI in each group. *P* values < 0.05 were considered statistically significant.

## 3. Results

### 3.1. Plasma IL-18 Concentrations

**Figure 1:**
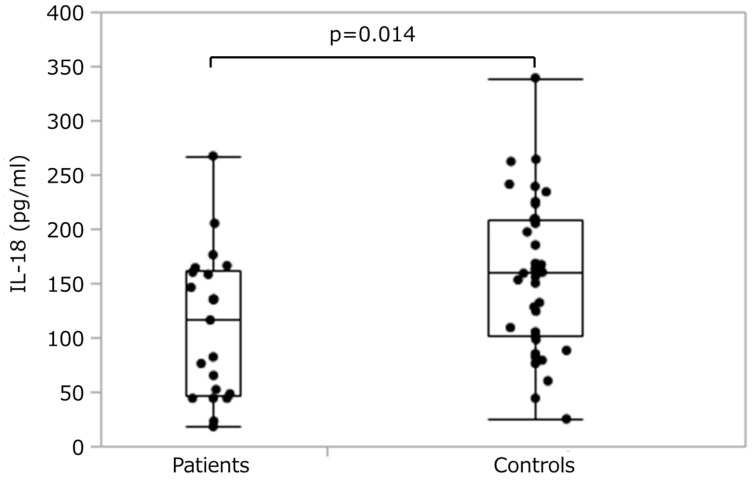
Plasma IL-18 concentrations by group. Box-and-whisker plot shows range, interquartile range, and median values.

Figure 1 shows plasma IL-18 concentrations for each group. The patient group showed significantly (*p* = 0.014) lower concentrations (median 116 pg/mL, range 18–267 pg/mL) than controls (median 160 pg/mL, range 25–339 pg/mL).

### 3.2. Correlations between Plasma IL-18 Concentration and Body Mass Index (BMI)

**Figure 2:**
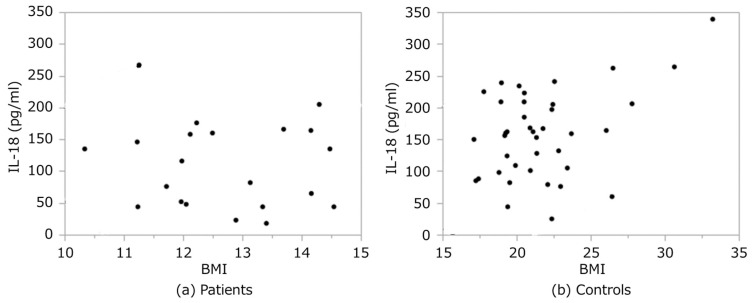
Correlations between plasma IL-18 concentration and BMI in (**a**) patients and (**b**) controls. BMI: body mass index.

Plasma IL-18 concentrations correlated moderately with BMI in the control group (r = 0.41, *p* = 0.01). However, no such correlations were observed in patients (r = −0.12, *p* = 0.62) (Figure 2).

## 4. Discussion

The lack of correlation between plasma IL-18 levels and BMI in patients with AN suggests that plasma IL-18 concentrations are affected not only by nutritional status but by an unknown pathophysiologic factor in these patients. Increased levels of IL-18 in plasma have been observed in patients with schizophrenia and moderate to severe depression [14], so a psychiatric comorbidity may have confounded plasma IL-18 levels in patients with AN.

When exposing mice to chronic stress, IL-18 expression is increased in the basolateral amygdala (BLA). IL-18 knock-out mice show tolerance to chronic stress, through a decline of vasopressin and oxytocin levels in the BLA, caused by suppression of phosphorylated nuclear factor-κB induced from IL-18 [15,16]. Plasma IL-18 concentrations increase in people with major depressive disorder, followed by decreases in endogenous opioid levels in the left amygdala and right hypothalamus and increases in the right ventral tegmental area [17,18,19]. These previous reports suggest that IL-18 is involved in the brain’s response to sadness and chronic stress.

Patients with AN often show chronic depression [20,21]; however, they frequently resist receiving treatment and tend to experience chronic AN [1,22]. The cause of decreased IL-18 levels in patients with AN cannot be explained only by malnutrition, and although the causal relationship is unknown, it is possible that these decreased levels persist because of the tendency of AN to become chronic.

Our research had a small sample size, so a replication study and subtype-sensitive research, with larger samples, are needed. Pharmacologic and nutritional effects on plasma IL-18 levels cannot be excluded. The time of day for blood sampling from control participants was not fixed, so diurnal effects of plasma IL-18 cannot be disregarded. Because the linear association of IL-18 levels in plasma and in cerebrospinal fluid (CSF) has not been proven, levels of IL-18 in CSF should be measured. However, lumbar punctures may be unsafe in patients with AN, who tend to have hemorrhagic issues. Thus we did not determine CSF levels. It is unclear why plasma IL-18 levels in patients with AN did not correlate with BMI levels. Pathophysiologic factors in patients with AN other than low body weight or malnutrition should be examined. Moreover, future prospective and longitudinal studies focused on disease duration, measuring IL-18 at different stages of AN including recovered patients, psychosocial factors and determining IL-18 and endogenous opioid levels in CSF may be needed. Our current results lead us to hypothesize that the decline of plasma IL-18 levels in patients with AN may affect disease chronicity.

## Figures and Tables

**Table 1:** Demographic data of participants.

	Patients	Controls	*p*-Value
Numbers	21	39	
Age (years)	30.14 ± 7.89	28.03 ± 7.37	*p* = 0.30
Education (years)	14.38 ± 2.11	15.72 ± 1.64	*p* < 0.01
BMI (kg/m^2^)	12.70 ± 1.24	21.71 ± 3.52	*p* < 0.01

BMI: body mass index. All values are mean ± S.D. Age, education, and BMI were compared between groups using Student’s *t*-test.

## References

[1] Zipfel S., Giel K.E., Bulik C.M., Hay P., Schmidt U. (2015). Anorexia nervosa: Aetiology, assessment, and treatment. Lancet Psychiatry.

[2] Nova E., Gomez-Martinez S., Morande G., Marcos A. (2002). Cytokine production by blood mononuclear cells from in-patients with anorexia nervosa. Br. J. Nutr..

[3] Nagata T., Kiriike N., Tobitani W., Kawarada Y., Matsunaga H., Yamagami S. (1999). Lymphocyte subset, lymphocyte proliferative response, and soluble interleukin-2 receptor in anorexic patients. Biol. Psychiatry.

[4] Allende L.M., Corell A., Manzanares J., Madruga D., Marcos A., Madrono A., Lopez-Goyanes A., Garcia-Perez M.A., Moreno J.M., Rodrigo M. (1998). Immunodeficiency associated with anorexia nervosa is secondary and improves after refeeding. Immunology.

[5] Brambilla F., Bellodi L., Brunetta M., Perna G. (1998). Plasma concentrations of interleukin-1 beta, interleukin-6 and tumor necrosis factor-alpha in anorexia and bulimia nervosa. Psychoneuroendocrinology.

[6] Monteleone P., Maes M., Fabrazzo M., Tortorella A., Lin A., Bosmans E., Kenis G., Maj M. (1999). Immunoendocrine findings in patients with eating disorders. Neuropsychobiology.

[7] Brambilla F., Monti D., Franceschi C. (2001). Plasma concentrations of interleukin-1-beta, interleukin-6 and tumor necrosis factor-alpha, and of their soluble receptors and receptor antagonist in anorexia nervosa. Psychiatry Res..

[8] Nagata T., Tobitani W., Kiriike N., Iketani T., Yamagami S. (1999). Capacity to produce cytokines during weight restoration in patients with anorexia nervosa. Psychosom. Med..

[9] Solmi M., Veronese N., Favaro A., Santonastaso P., Manzato E., Sergi G., Correll C.U. (2015). Inflammatory cytokines and anorexia nervosa: A meta-analysis of cross-sectional and longitudinal studies. Psychoneuroendocrinology.

[10] Okamura H., Tsutsui H., Komatsu T., Yutsudo M., Hakura A., Tanimoto T., Torigoe K., Okura T., Nukada Y., Hattori K. (1995). Cloning of a new cytokine that induces IFN-γ production by T cells. Nature.

[11] Okamura H., Nagata K., Komatsu T., Tanimoto T., Nukata Y., Tanabe F., Akita K., Torigoe K., Okura T., Fukuda S. (1995). A novel costimulatory factor for gamma interferon induction found in the livers of mice causes endotoxic shock. Infect. Immun..

[12] Lebel-Binay S., Berger A., Zinzindohoué F., Cugnenc P., Thiounn N., Fridman W.H., Pagès F. (2000). Interleukin-18: Biological properties and clinical implications. Eur. Cytokine Netw..

[13] American Psychiatric Association (2013). Diagnostic and Statistical Manual of Mental Disorders (DSM-5®).

[14] Alboni S., Cervia D., Sugama S., Conti B. (2010). Interleukin 18 in the CNS. J. Neuroinflamm..

[15] Kim T.-K., Kim J.-E., Choi J., Park J.-Y., Lee J.-E., Lee E.-H., Lee Y., Kim B.Y., Oh Y.J., Han P.-L. (2017). Local Interleukin-18 System in the Basolateral Amygdala Regulates Susceptibility to Chronic Stress. Mol. Neurobiol..

[16] Yamamoto Y., Tanahashi T., Katsuura S., Kurokawa K., Nishida K., Kuwano Y., Kawai T., Teshima-Kondo S., Chikahisa S., Tsuruo Y. (2010). Interleukin-18 deficiency reduces neuropeptide gene expressions in the mouse amygdala related with behavioral change. J. Neuroimmunol..

[17] Prossin A.R., Koch A.E., Campbell P.L., Barichello T., Zalcman S.S., Zubieta J.K. (2016). Experimental sadness induces relevant interactions between central endogenous opioid activation and plasma IL-18 concentrations in depressed volunteers. Mol. Psychiatry.

[18] Prossin A.R., Koch A.E., Campbell P.L., McInnis M.G., Zalcman S.S., Zubieta J.-K. (2011). Association of Plasma Interleukin-18 Levels with Emotion Regulation and μ-Opioid Neurotransmitter Function in Major Depression and Healthy Volunteers. Biol. Psychiatry.

[19] Prossin A.R., Koch A.E., Campbell P.L., Barichello T., Zalcman S.S., Zubieta J.K. (2016). Acute experimental changes in mood state regulate immune function in relation to central opioid neurotransmission: A model of human CNS-peripheral inflammatory interaction. Mol. Psychiatry.

[20] Casper R.C. (1998). Depression and eating disorders. Depress. Anxiety.

[21] Mattar L., Huas C., Duclos J., Apfel A., Godart N. (2011). Relationship between malnutrition and depression or anxiety in Anorexia Nervosa: A critical review of the literature. J. Affect. Disord..

[22] Kaye W.H., Fudge J.L., Paulus M. (2009). New insights into symptoms and neurocircuit function of anorexia nervosa. Nat. Rev. Neurosci..

